# Organophosphorus Chemistry for the Synthesis of Dendrimers

**DOI:** 10.3390/molecules171113605

**Published:** 2012-11-16

**Authors:** Anne-Marie Caminade, Régis Laurent, Maria Zablocka, Jean-Pierre Majoral

**Affiliations:** 1 CNRS, LCC (Laboratoire de Chimie de Coordination), 205 route de Narbonne, BP44099, F-31077 Toulouse Cedex 4, France; Email: rlaurent@lcc-toulouse.fr (R.L.); majoral@lcc-toulouse.fr (J.-P.M.); 2 Université de Toulouse, UPS, INPT, F-31077 Toulouse Cedex 4, France; 3 Centre of Molecular and Macromolecular Studies, The Polish Academy of Sciences, Sienkiewicza 112, 90363 Lodz, Poland; Email: zabloc@cbmm.lodz.pl

**Keywords:** dendrimers, phosphorus, hydrazone, phosphine, phosphonate

## Abstract

Dendrimers are multifunctional, hyperbranched and perfectly defined macromolecules, synthesized layer after layer in an iterative manner. Besides the nature of the terminal groups responsible for most of the properties, the nature of the internal structure, and more precisely of the branching points, is also of crucial importance. For more than 15 years, we have demonstrated that the presence of phosphorus atom(s) at each branching point of the dendrimeric structure is particularly important and highly valuable for three main reasons: (i) the versatility of phosphorus chemistry that allows diversified organochemistry for the synthesis of dendrimers; (ii) the use of ^31^P-NMR, which is a highly valuable tool for the characterization of dendrimers; (iii) some properties (in the fields of catalysis, materials, and especially biology), that are directly connected to the nature of the internal structure and of the branching points. This review will give an overview of the methods of synthesis of phosphorus-containing dendrimers, as well on the ways to graft phosphorus derivatives as terminal groups, with emphasis on the various roles played by the chemistry of phosphorus.

## 1. Introduction

**Scheme 1:**
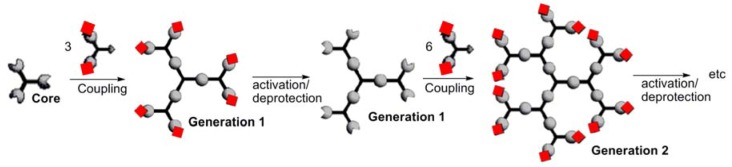
The principle of the divergent synthesis of dendrimers.

Dendrimers [1] have an aesthetic structure constituted of branching units emanating radially from a central core. They are synthesized step-by-step in an iterative fashion. Each time the number of terminal groups is multiplied, a new generation is created. Due to this highly controlled synthesis, dendrimers offer a perfect modularity of size (a few nanometers), functionality, and solubility, mainly depending on the type of their terminal groups. Scheme 1 displays the principles of the divergent process, most generally used for the synthesis of dendrimers.

Among all types of dendrimers [2], phosphorus-containing dendrimers [3] that have one phosphorus atom at each branching point, play an important role, with applications ranging from catalysis [4], materials [5], and even biology/nanomedicine [6]. This review will focus on our work, emphasizing the role of phosphorus [7]. It will be organized depending on the type of reactions that will occur on phosphorus atoms, whatever their location. All the other reactions of phosphorus-containing dendrimers, but not occurring on the P atoms will not be displayed, except if they are a necessary pathway towards the chemistry of phosphorus, or for the grafting of phosphorus entities.

## 2. Substitution Reactions on P-Cl Functions for the Synthesis and Functionalization of Dendrimers

**Scheme 2:**
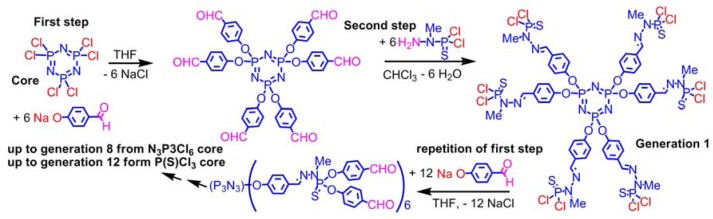
The most important method of synthesis of phosphorus dendrimers.

Our first and main method of synthesis of phosphorus dendrimers [8] consists in the repetition of two quantitative reactions, the first step being the nucleophilic substitution of Cl by 4-hydroxybenzaldehyde in basic conditions. The second step is the condensation of the aldehydes with the dichlorophosphothiohydrazide. This compound is also issued from the organic chemistry of phosphorus (substitution of one Cl of P(S)Cl_3_ with methylhydrazine, at low temperature). This second step generates P-Cl_2_ functions suitable to perform again substitutions with HOC_6_H_4_CHO (Scheme 2).

**Figure 1:**
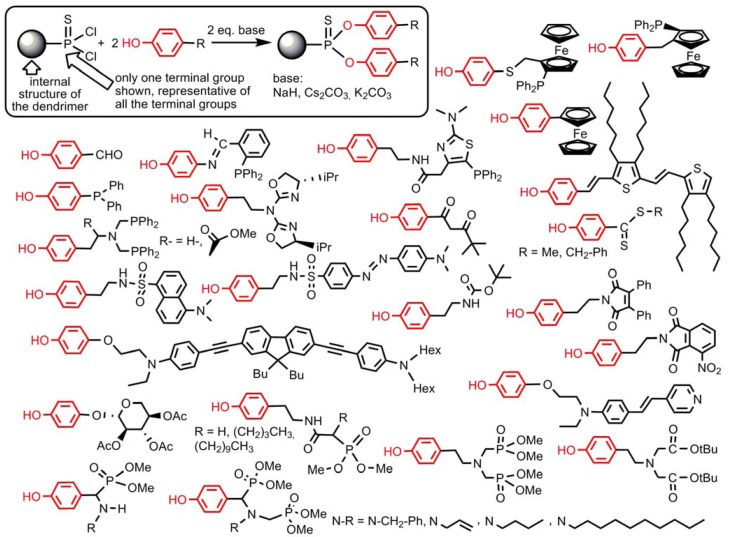
Functionalized phenols that have been grafted to dendrimers ended by P(S)Cl_2_ functions.

This method is very powerful and has been carried out up to generation 8 starting from N_3_P_3_Cl_6_ [9], and to generation 12 (the highest generation ever synthesized for any type of dendrimers) from P(S)Cl_3_ [10]. The substitution reaction of P-Cl by phenols is quantitative in most cases, using <5% excess of reagents. It is particularly powerful for the functionalization of the surface of dendrimers, with variously functionalized phenols depending on the desired properties. Several examples are displayed in Figure 1. One can cite in particular the aldehyde (for the elaboration of DNA chips) [11], various ferrocenes (for studying electrochemical properties [12], evolution of chirality [13], and for catalysis [14]), various ligands suitable for catalysis such as derivatives of triphenylphosphine [15] (also precursors of phosphoniums [16]), thiazolylphosphines [17], iminophosphines [18], diphosphines [19], diketones [20], or azabis-oxazolines [21], dithioesters for thioacylation reactions (R = Me) [22] or as precursors of star polymers (R = CH_2_Ph) [23]. Several fluorophores such as maleimide derivatives [24], dansyl derivatives [25] and also dabsyl dyes and protected tyramine [26], or fluorophores having two-photon absorption (TPA) properties [27], with eventually interchromophoric activities [28], or third order non-linear properties [29], have been grafted thanks to the reactivity of phenols, as well as D-xylose derivatives [30], phosphonates as precursors of anti-HIV derivatives [31], and azabisphosphonates (and isosteric carboxylic esters analogues [32]) precursors of symmetrical [33] or non-symmetrical [34] azabisphosphonic salts having important biological properties. In all cases, ^31^P-NMR is a precious tool for characterizing these dendrimers and the achievement of the reactions [35].

**Figure 2:**
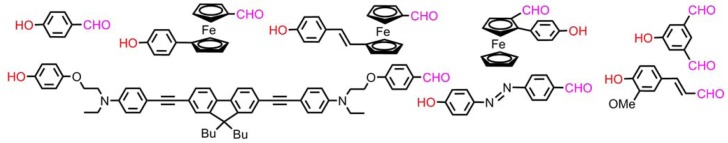
Some phenol aldehydes used instead of HOC_6_H_4_CHO for the synthesis of dendrimers.

The substitution reactions with phenols are also usable for varying the internal structure of dendrimers, by replacing 4-hydroxybenzaldehyde by other phenol aldehydes (Figure 2). Among them, one can cite the possibility to have various ferrocenes [12,36,37], longer branches [38], fluorophores [39], azobenzenes [40], or dialdehydes [41] for multiplying rapidly the number of terminal functions.

**Scheme 3:**
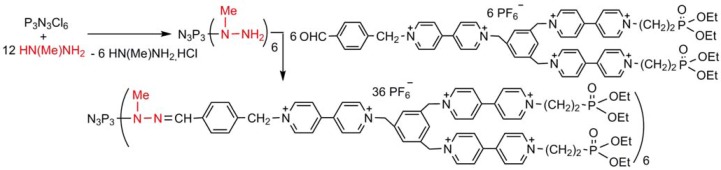
Synthesis of a small dendrimer via substitutions with methylhydrazine.

We have also attempted to use the substitution reactions of hydrazines for the synthesis of dendrimers [42,43]. The most recent example is shown on Scheme 3 [44]. However, none of them give perfectly quantitative yields, and they have been carried out only up to the first generation. 

**Figure 3:**
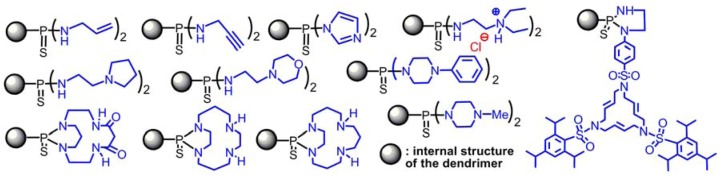
Functionalization of the surface of dendrimers by amino derivatives (only one function is shown, representative of all the terminal groups).

On the contrary, the substitution reactions with amines are very powerful for functionalizing the surface of dendrimers, starting from P(S)Cl_2_ end groups (Figure 3). Among them, one can cite in particular allyl and propargyl amines [45], and also diethylethylenediamine, which affords in a single step water-soluble dendrimers; HCl generated by the substitution reaction is trapped by the tertiary amine [46]. Water-soluble dendrimers [47] have important biological properties [48], but those ones possess interesting properties both in the fields of materials (for the elaboration of nano-tubes [49] and micro-capsules [50], of highly sensitive DNA chips [51]), and biology (transfection agents [46], anti-prion agents [52], anti-aggregation agent of Alzheimer peptides [53]). Other types of diamines, such as morpholine or piperidine derivatives have also been used [54]. In another example, both Cl linked to the same P react with a single diamine, creating a diazaphospholane cycle. This cycle can be obtained from various macrocycles [55], or can be linked to a macrocycle that is able to complex Pd^0^ [56] or Pt^0^ [57], to create nanoparticles of these metals, and even to organize them in dendritic networks [58].

**Figure 4:**
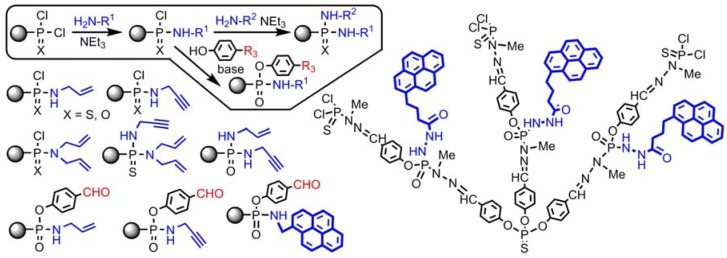
Multifunctionalization of the surface of dendrimers, and of the internal structure.

The reactions with amines are also suitable to perform clean monosubstitutions on each P(X)Cl_2_ (X = S, O) end group. The reaction is regiospecific, but not enantioselective. The monosubstitution with amines leads to dendrimers with two, three, and even four unique functional groups on each chain end [59]. The second substitution can be performed with another amine, but also with phenols, in particular HOC_6_H_4_CHO, leading to dendrimers having functions in the internal structure [60] (Figure 4).

## 3. Diverse Ways for Grafting Phosphorus Entities as Terminal Groups

**Scheme 4:**
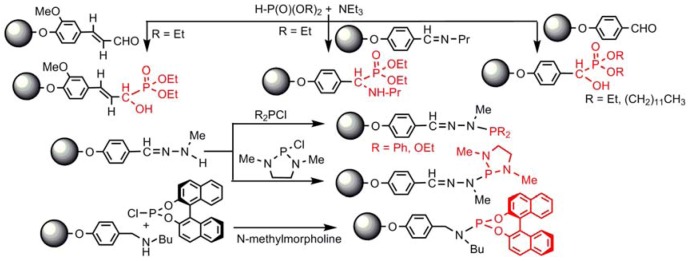
Functionalization of terminal groups by direct reaction of phosphorus derivatives.

Diverse reactions have been performed to graft phosphorus derivatives as terminal groups of dendrimers. They can be divided into two types: those occurring on phosphorus, and those occurring on a function linked to phosphorus. In the first case, two different types of reactions have been performed: the addition of P-H onto unsaturated bonds such as aldehydes and imines [61], and the substitution reactions of P-Cl with N-H functions, from hydrazones [62] or amines [63] (Scheme 4).

**Scheme 5:**
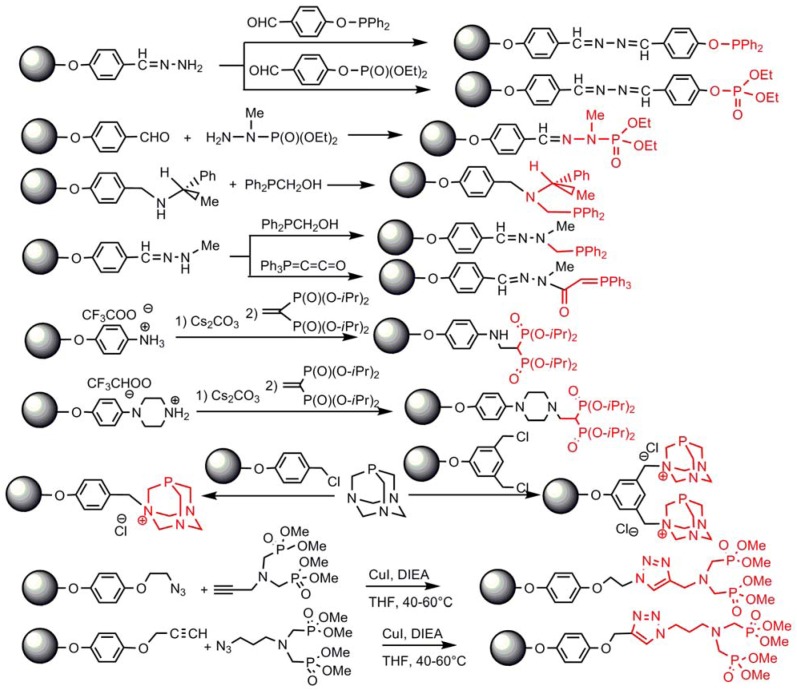
Other types of reactions for the grafting of phosphorus derivatives as terminal groups.

Various phosphorus derivatives, in particular phosphines and phosphonates or phosphates, have been grafted to the terminal groups of dendrimers essentially through condensation reactions, addition reactions and “click” reactions. The condensation reaction of hydrazones with aldehydes has afforded phosphites, phosphates or aminophosphates as terminal groups [61], whereas the condensation with Ph_2_PCH_2_OH on chiral amines (or hydrazone [64]) has led to chiral phosphines [65]. Addition reactions of amino groups onto unsaturated bonds have led to the grafting of ylides [61], or gem-bisphosphonates [66]. Alkylation of one nitrogen of PTA (phosphatriazaadamantane) has led to the grafting of one [67] or two [68] PTA per terminal function. Finally, the “click” reaction (azides with alkynes) has led to the grafting of azabisphosphonate groups [69] (Scheme 5).

## 4. Staudinger Reactions and Subsequent Reactions

**Scheme 6:**
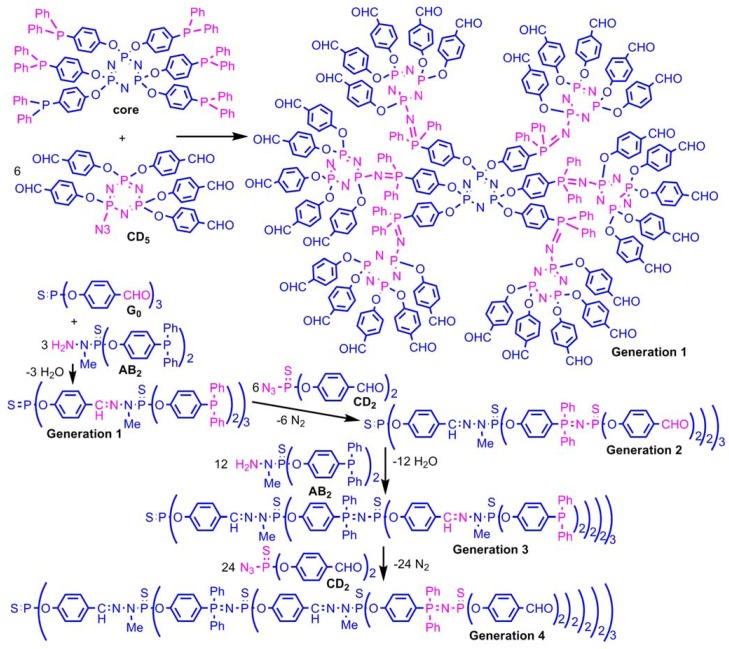
Two methods of synthesis of dendrimers by Staudinger reactions.

The Staudinger reaction of phosphines with azides creates P=N functions; which are generally sensitive to hydrolysis. However, if the P=N function is conjugated, its stability is largely increased. Thus, instead of using organic azides, we have used thiophosphoryl azides, to generate P=N-P=S functions (or eventually P=N-P=N functions when using azides linked to the cyclotriphosphazene, as shown in the following scheme). We have synthesized several types of monomers to use alternatively the condensation reaction (aldehyde with hydrazine) and the Staudinger reaction. These monomers comprise either phosphines and hydrazine, or aldehydes and azide, in a 2/1 [70] or 5/1 ratio [71], eventually in combination [72]. Using these monomers allows a rapid multiplication of the number of terminal groups, and creates a new generation at each step and not every two steps as usual. This method of synthesis is also compatible with the first one mentioned in Scheme 2 (Scheme 6) [73].

**Scheme 7:**
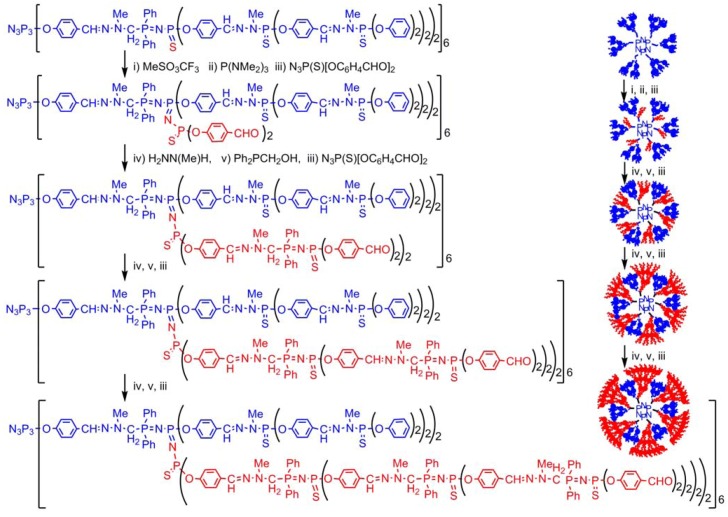
Reactivity of the P=N-P=S linkages and growing of new branches inside the dendrimer.

The compatibility of the Staudinger reaction with the condensation reaction allows having P=N-P=S linkages selectively at one or two layers. The P=S groups linked to a P=N group have distinguishable properties compared to the other P=S groups, due to a delocalized form ^+^P-N=P-S^−^, with a negative charge on S, which renders it sensitive to alkylation reactions [74] using various triflates [75] whereas the other P=S groups do not react. It is also suitable for the complexation of gold [76]. The alkylation induces a weakening of the PS bond, which can be cleaved using a nucleophilic phosphine such as P(NMe_2_)_3_. This reaction generates tricoordinated phosphorus atoms (P^III^) at specific layers of the internal structure, that can be used for alkylation reactions, and can undergo Staudinger reactions creating P=N-P=N-P=S linkages [77]. The presence of aldehydes inside the dendrimers allows either the step-by-step growing of new branches [78] (Scheme 7), or the grafting of dendrons, leading to highly sophisticated dendrimeric structures [79], still unprecedented for any type of dendrimers, but also the grafting of new functions such as fluorescent groups [80], or zwitter-ions [81]. 

**Scheme 8:**
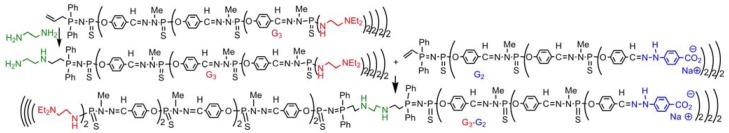
Example of synthesis of a Janus dendrimer, thanks to the presence of P=N-P=S linkages.

The P=N-P=S linkage is also able to activate vinyl groups linked to the phosphazene, and used as core of dendrons (dendritic wedges). Different types of amines were used for Michael-type additions, suitable for grafting together by their core two dendrons which differ by their terminal functions such as nitrile, amine or phosphine [82] but also amine and carboxylate [83], leading to “Janus” dendrimers [84] (Scheme 8).

## 5. Wittig and Horner-Wadsworth-Emmons Reactions

**Scheme 9:**
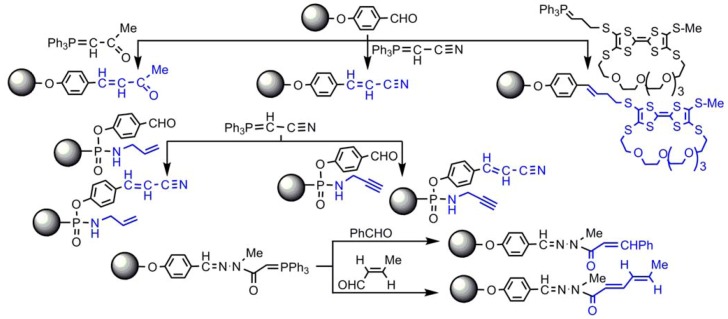
Use of the Wittig reaction for the functionalization of the surface of dendrimers.

We have used this classical phosphorus reaction for the functionalization of the terminal groups of dendrimers, starting from the aldehyde functions. The Wittig reaction was used in particular for the grafting of ketone and nitrile [45], or tetrathiafulvalene (TTF) derivatives, including one with a macrocyclic substituent, suitable for the electrochemical sensing of Ba^2+^ [85]. The Wittig reaction was also applied when only half of the terminal groups were aldehydes [59], or ylides [61] (Scheme 9). 

**Scheme 10:**
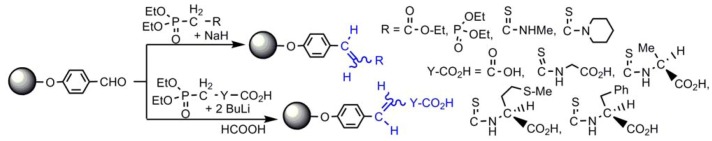
Horner-Wadsworth-Emmons reaction for the grafting of aminoacids.

The Horner-Wadsworth-Emmons reaction has been applied to the aldehyde terminal functions, affording predominantly the E isomers, in particular for the grafting of aminoacids [86] (Scheme 10).

## 6. Cleavage of P-OR Bonds

**Scheme 11:**
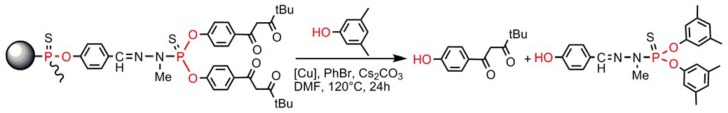
Cleavage of P-OR bonds in catalysis conditions.

In the course of our studies about the physico-chemical properties of phosphorus dendrimers, we have studied their thermal stability, and discovered that in many cases the first mass loss corresponds to the peeling of the surface, thus to the cleavage of the P-OR terminal groups at high temperature (above 200 °C for the least stable, but generally above 350 °C) [87]. Such cleavage has been also observed in the case of diketone terminal groups used for the complexation of copper, then for catalyzing diarylether formation at 120 °C. The efficiency of the catalysis was found independent of the generation of the dendrimer, and no reuse was possible, contrarily to what we had observed in all previous examples of catalysis [88]. Studying in details the reaction media after catalysis, we found a large amount of the monomer, resulting from the cleavage of the surface of the dendrimers. It must be noted that the cleavage is due to the catalysis, since the dendrimer is recovered intact in the same conditions, but in the absence of metal (Scheme 11) [17]. 

**Scheme 12:**
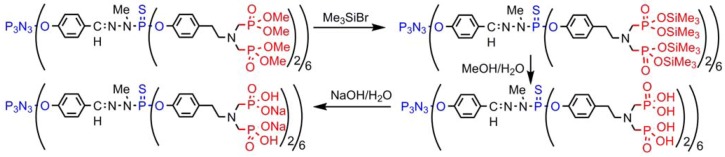
Cleavage of P-OMe bonds while preserving P-OAr bonds.

The dendrimers ended by azabisphosphonate groups are not easily soluble in water, thus we tried to obtain phosphonic acid instead of phosphonate terminal groups. For this purpose, the first step is the reaction with bromotrimethylsilane, which generates P-O-SiMe_3_ groups, subsequently hydrolyzed. The last step is the reaction with NaOH (Scheme 12), affording water-soluble dendrimers [89], which possess very important biological properties [90], in particular towards the human immune system [91], as anti-inflammatory drug [92], and against rheumatoid arthritis [93].

## 7. Conclusions

A large panel of organophosphorus reactions has been used for the synthesis of phosphorus-containing dendrimers. Besides the efficiency of these reactions, the simplicity of characterization of these large compounds by ^31^P-NMR has to be emphasized. Indeed, even highly sophisticated structures can be totally analyzed by ^31^P-NMR [78]. It must be emphasized also that the presence of phosphorus leads to unprecedented properties, particularly in the fields of catalysis, materials, and biology.

Besides our work, which has been largely displayed in this review, a few other groups have reported the synthesis of phosphorus-containing dendrimers. We have to mention in particular the pioneering work made by R. Engel (polyphosphonium dendrimers) [94], M. J. Damha (nucleic acid dendrimers) [95], and D. L. DuBois (small polyphosphines) [96]. Later on, large polyphosphine dendrimers have been proposed by A. K. Kakkar [97], and also thiophosphate dendrimers by G. M. Salamonczyk [98] based on the use of phosphoramidite reagents. Taken all together, these researches demonstrate the rich diversity of the chemistry of phosphorus, even when applied to nano-objects such as dendrimers.
